# Multiple-Hit Hypothesis in Parkinson’s Disease: LRRK2 and Inflammation

**DOI:** 10.3389/fnins.2020.00376

**Published:** 2020-04-28

**Authors:** Diego Cabezudo, Veerle Baekelandt, Evy Lobbestael

**Affiliations:** Laboratory for Neurobiology and Gene Therapy, Department of Neurosciences, KU Leuven, Leuven, Belgium

**Keywords:** LRRK2, Parkinson’s disease, inflammation, neuroinflammation, IBD

## Abstract

The multiple hit hypothesis for Parkinson’s disease (PD) suggests that an interaction between multiple (genetic and/or environmental) risk factors is needed to trigger the pathology. Leucine-Rich Repeat Kinase 2 (LRRK2) is an interesting protein to study in this context and is the focus of this review. More than 15 years of intensive research have identified several cellular pathways in which LRRK2 is involved, yet its exact physiological role or contribution to PD is not completely understood. Pathogenic mutations in LRRK2 are the most common genetic cause of PD but most likely require additional triggers to develop PD, as suggested by the reduced penetrance of the LRRK2 G2019S mutation. LRRK2 expression is high in immune cells such as monocytes, neutrophils, or dendritic cells, compared to neurons or glial cells and evidence for a role of LRRK2 in the immune system is emerging. This has led to the hypothesis that an inflammatory trigger is needed for pathogenic LRRK2 mutations to induce a PD phenotype. In this review, we will discuss the link between LRRK2 and inflammation and how this could play an active role in PD etiology.

## Introduction

Parkinson’s disease (PD) is the most common motor neurodegenerative disorder, estimated to affect about 7 million people worldwide. Pathologically, it is characterized by the degeneration of the dopaminergic neurons in the substantia nigra (SN), the aggregation of α-synuclein (αSYN) in cytoplasmic inclusions named Lewy Bodies, and neuroinflammation. The first evidence for neuroinflammation in PD was the discovery of human leukocyte antigen D-related (HLA-DR)-positive microglia in the SN of PD patients by McGeer et al. (1988a). Since then, intensive research has focused on understanding the extent and contribution of neuroinflammation to the progression of PD. The microgliosis that takes place in PD brain is accompanied by astrogliosis and an increase in the expression of inflammatory cytokines, chemokines, and prostaglandins (Mogi et al., 1994, 1996; Hunot et al., 1999; Teismann and Schulz, 2004; Teismann, 2012). Additionally, immunoglobulins G (IgGs) surround the Lewy Bodies and dopaminergic neurons, which points to the contribution of both the innate and adaptive immune system to neuroinflammation in PD (Orr et al., 2005).

## Inflammation and Parkinson’s Disease

Although the etiology of PD is not well understood, it is generally believed that the immune system plays an active role, and that the neuroinflammation observed in the patient’s brain might not only be a consequence of the ongoing neurodegeneration, as initially hypothesized (Tansey and Goldberg, 2010). The contribution of neuroinflammation to the pathology could explain the selective neuronal death in PD. Neuroinflammation induces the accumulation of cytokines and reactive oxygen species (ROS) in the brain, to which the dopaminergic neurons from the SN are particularly susceptible (reviewed in Tansey and Goldberg, 2010; Dias et al., 2013). Additionally, neuroinflammatory effects might be more pronounced in the SN, as this brain region displays the highest density of microglia, which are the brain resident macrophages, in the brain (Yang et al., 2013).

Genome-wide association studies (GWAS) found a connection between variations in the HLA locus and sporadic PD, thereby identifying the immune system as a contributor to PD susceptibility (Hamza et al., 2010; Saiki et al., 2010; Nalls et al., 2011; Holmans et al., 2013; Wissemann et al., 2013; Zhang et al., 2017). As opposed to what was believed in the past, the brain is not a completely immune privileged organ. Inflammatory events taking place outside the central nervous system (CNS) can communicate with the microglia and alter their activation state leading or contributing to neuroinflammation (McManus and Heneka, 2017). Communication between the periphery and the CNS is an important step for the peripheral immune system to initiate a harmful response in the brain. Peripheral cytokines and other inflammatory mediators can act on the perivascular macrophages and macrophages from the circumventricular organs of the brain, in which the blood brain barrier (BBB) is more permeable (Lacroix et al., 1998). T cells, B cells, natural killer cells and dendritic cells are present in other permeable regions like the choroid plexus and the meninges and may serve as a bridge to the brain (Korin et al., 2017). More specifically for enteric inflammation, the inflammatory mediators can act on the neurons forming the afferent vagus nerve, hence influencing other regions of the CNS (Perry and Teeling, 2013). Additionally, disruption of the BBB has been described in pathological conditions and has been extensively reported in PD patients (Maiuolo et al., 2018; Sweeney et al., 2018; Fuzzati-Armentero et al., 2019). The opening of the BBB permits the infiltration of immune cells into the brain parenchyma, which can exacerbate the neuroinflammatory environment of the diseased brain. This is in line with the T cell infiltration that is consistently found in the SN of patients and PD models (McGeer et al., 1988b; Brochard et al., 2008).

**FIGURE 1:**
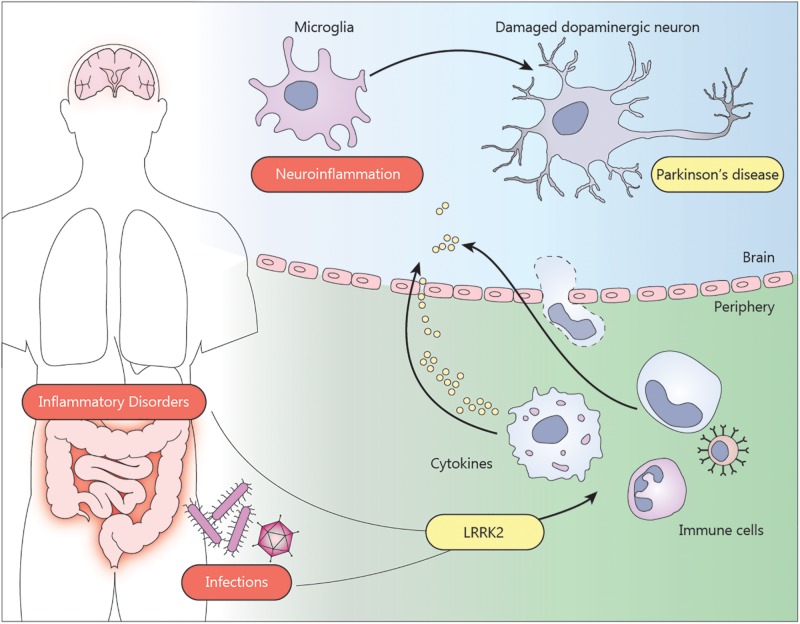
Environmental factors such as inflammatory bowel disease or infections can trigger neuroinflammation and contribute to the pathogenesis of Parkinson’s disease. The presence of LRRK2 mutations exacerbate the pro-inflammatory state of the immune cells from the periphery. Infiltration of monocytes, T cells or cytokines through the blood brain barrier can induce the activation of microglia in the brain. The neuroinflammatory environment affects the dopaminergic neurons in the substantia nigra, contributing to the neurodegeneration.

The communication between the periphery and the CNS implies that infections or inflammatory events can act as environmental factors triggering or contributing to the PD pathogenesis (Figure 1). This idea is supported by several epidemiological studies. A first hint came from the Spanish flu pandemic in 1918. Affected people were reported to develop transient parkinsonian symptoms the month after infection (Ravenholt and Foege, 1982; Toovey et al., 2011). More interesting, an increased risk to develop PD was found in a cohort of patients with tuberculosis (Shen et al., 2016), vermiform appendix (Killinger et al., 2018), or inflammatory bowel disease (IBD) (Wan et al., 2018; Park et al., 2019; Weimers et al., 2019; Zhu et al., 2019). Furthermore, IBD patients treated with anti-tumor necrosis factor (anti-TNF) therapy showed no increased risk for PD, further supporting a contributive role of inflammation in PD etiology (Peter et al., 2018). This was not the first time an anti-inflammatory treatment was proposed to protect against neurodegeneration. Chronic treatment with non-steroidal anti-inflammatory drugs (NSAIDs) was linked to a reduced predisposition to develop PD (Chen et al., 2005; Gagne and Power, 2010), although several other studies failed to confirm these results (Driver et al., 2011; Ren et al., 2018; Poly et al., 2019).

## LRRK2 and Neuroinflammation

Approximately 10% of all PD cases have a monogenic origin, with mutations in genes encoding for α-synuclein (SNCA), Leucine-Rich Repeat Kinase 2 (LRRK2), Parkin, PTEN-induced putative kinase 1 (PINK1), or DJ1 as the most studied examples (Nuytemans et al., 2010). These disease-causing mutations have indicated key cellular processes in PD etiology. Nevertheless, and despite being the most common PD-linked gene, the exact role of LRRK2 still remains unclear. Below, we will discuss evidence supporting the idea that LRRK2 constitutes a strong link between inflammation and PD.

LRRK2 was first described in 2004 as a PD-related gene. The most frequent G2019S mutation accounts for 4% of the familial and 1% of the sporadic PD cases (Domingo and Klein, 2018). Most of the pathogenic LRRK2 mutations enhance kinase activity, which has been linked to pathological phenotypes in neurons (Korecka et al., 2019). LRRK2 has been linked to several cellular processes including mitochondrial function, endocytosis, vesicle trafficking, autophagy, and processes at the *trans*-Golgi network (reviewed in Wallings et al., 2015; Albanese et al., 2019; Berwick et al., 2019). More mechanistic insight in these functions came from the identification of several Rab proteins as *bona fide* LRRK2 substrates (Steger et al., 2016; Fujimoto et al., 2017; Liu et al., 2018; Rivero-Ríos et al., 2019). These small GTPases are regulators of membrane trafficking and are also involved in cellular processes essential for immune cell activity such as phagocytosis, exocytosis, and antigen presentation (reviewed in Prashar et al., 2017; Wallings and Tansey, 2019). This is in line with the emerging evidence pointing to LRRK2 as a modulator of inflammation through a role in immune cells both in the CNS and the periphery.

Several studies have reported the dysregulation of inflammatory events by LRRK2 *in vivo.* Already in 2009, Lin et al. (2009) reported an increase in microgliosis and astrogliosis in A53T αSYN transgenic mice in the presence of LRRK2 G2019S. However, no effect of the G2019S mutation could be observed in microglia in a different transgenic αSYN model (Daher et al., 2012). In 2015, Daher et al. (2015) reported an increased activation of microglia in the SN of a G2019S LRRK2 transgenic rat after recombinant adeno-associated viral vector (rAAV)-mediated αSYN overexpression. This increase in neuroinflammation was accompanied by a more pronounced neurodegeneration and could be abolished by the inhibition of LRRK2 kinase activity. Recently, another study showed increased expression of CD68 in microglia from G2019S LRRK2 mice injected with recombinant αSYN fibrils, as well as increased expression of pro-inflammatory markers such as IL-6, TNFα and C1qa and astroglial markers like Vim, CD44 and Cxcl10 (Bieri et al., 2019). In addition, a physiological role for WT LRRK2 in neuroinflammation is supported by studies using LRRK2 knock out (KO) models. Genetic ablation of LRRK2 was reported to protect against dopaminergic neurodegeneration induced by lipopolysaccharide (LPS), as well as against the neuroinflammation and neurodegeneration induced by rAAV-based overexpression of αSYN (Daher et al., 2014). LRRK2 KO animals displayed a reduced number of CD68 and iNOS positive cells and reduced myeloid cell activation as shown by the absence of a shift in morphology from ramified to amoeboid Iba1^+^-cells. The evidence that WT LRRK2 is not only involved in PD-related neuroinflammation is underlined by the finding that suppressing LRRK2 activity or expression is also protective against neuroinflammation after exposure to manganese (Chen et al., 2018) or HIV-1 Tat protein in an HIV-1 associated neurocognitive disorder (HAND) model *in vivo* (Puccini et al., 2015). Taken together, LRRK2 is considered as a pro-inflammatory agent in different neuroinflammatory animal models with increased LRRK2 kinase activity as a driver of inflammation.

## LRRK2 in Immune Cells

In order to understand the physiological and pathological function of LRRK2, it is essential to identify the cell types in which LRRK2 plays a major role. Microglia are the first barrier of the innate immune system in the brain. Therefore, most efforts to elucidate the function of LRRK2 in neuroinflammation have focused on this cell type. Reducing the expression or activity of LRRK2 in microglia was shown to reduce the levels of pro-inflammatory cytokines such as TNFa, IL6, IL-1b, or IL-10 *in vitro* (Kim et al., 2012; Moehle et al., 2012; Russo et al., 2015) as well as to enhance microglial motility induced by adenosine diphosphate (ADP) and fractalkine, characteristic of microglia in a non-reactive state (Choi et al., 2015; Ma et al., 2016). Contrarily, mutations enhancing LRRK2 activity such as G2019S or R1441G were reported to shift cultured microglia to a more pro-inflammatory phenotype (Gillardon et al., 2012; Caesar et al., 2014; Choi et al., 2015; Russo et al., 2018). In addition, elevated LRRK2 mRNA levels were found in human and rodent microglia and protein expression was induced in microglia after stimulation with LPS *in vitro* (Miklossy et al., 2006; Gillardon et al., 2012; Moehle et al., 2012). Despite the reported effects in microglia, the relevance of LRRK2 in this immune cell is still under debate. Several studies in wild-type and BAC LRRK2 transgenic mice could not identify LRRK2 expression in microglia (Biskup et al., 2006; Higashi et al., 2007b; Westerlund et al., 2008; Mandemakers et al., 2012). Similarly, *in situ* hybridization and immunohistochemical studies on brain sections from PD patients and healthy controls reported no expression of LRRK2 in microglia (Higashi et al., 2007a; Hakimi et al., 2011; Sharma et al., 2011; Dzamko et al., 2012, 2017). LRRK2 expression was also not detectable after LPS stimulation in microglia isolated from rodent brain (Kozina et al., 2018). These conflicting results might be due to *in vivo* vs. *in vitro* differences given the alterations in phenotype and expression profile when microglia are placed in culture (Schmid et al., 2009; Butovsky et al., 2014). Furthermore, immunohistochemical detection of microglia in brain is based either on morphological analyses or myeloid markers like Isolectin B4 (Miklossy et al., 2006; Moehle et al., 2012). Establishing LRRK2 expression in microglia is complicated since these markers are also expressed in peripheral monocytes, which express LRRK2 (Gardet et al., 2010; Thévenet et al., 2011; Cook et al., 2017) and are known to infiltrate the brain during disease progression.

As discussed above, CNS resident microglia might not be the only players in neuroinflammation observed in PD and other neurodegenerative diseases. Emerging evidence points to a key role for peripheral immune cells, but how changes in activation state of these cells contribute to neuroinflammation and neurodegeneration is one of the outstanding questions in the field. In this context, LRRK2 is a very attractive target since the highest LRRK2 expression is found in myeloid cells like monocytes, dendritic cells and neutrophils, and to a lower extent, in B and T cells (Gardet et al., 2010; Hakimi et al., 2011; Thévenet et al., 2011; Daher et al., 2015; Cook et al., 2017). LRRK2 mRNA and protein levels are upregulated in macrophages and leukocytes upon *in vitro* exposure to pathogens and inflammatory mediators such as IFN-γ, IFN-β, TNF-α, and IL-6 (Hakimi et al., 2011; Thévenet et al., 2011; Kuss et al., 2014). In addition, stimulation of Toll-like receptors was shown to result in phosphorylation, dimerization and membrane translocation of LRRK2, pointing to activation of its function (Schapansky et al., 2014). Interestingly, LRRK2 protein levels are increased in B cells, T cells (CD4+, CD8+, and T regulatory cells) and CD14+ as well as CD16+ monocytes in PD patients compared to healthy controls (Bliederhaeuser et al., 2016; Cook et al., 2017). Moreover, PD patient monocytes were reported to secrete more inflammatory cytokines, which positively correlated with LRRK2 expression in T cells from PD patients, but not healthy controls (Cook et al., 2017). A role for LRRK2 in peripheral immune cells is also supported by the higher levels of peripheral inflammatory cytokines in the sera of PD patients carrying LRRK2 G2019S, as well as in asymptomatic carriers of the mutation (Dzamko et al., 2016). Further evidence comes from a more recent study showing that mice overexpressing mutant but not WT LRRK2 displayed an exacerbated long-term response to treatment with the systemic inflammatory insult LPS that leads to neuroinflammation and neurodegeneration in the SN. Intriguingly, the enhanced neuroinflammation was induced by peripheral cytokines, rather than by dysfunctional microglia or infiltration of monocytes or T-cells (Kozina et al., 2018). An independent study confirmed that a single peripheral LPS dose causes significantly increased neuroinflammation in LRRK2 G2019S rats, but not in non-transgenic rats, 10 months after treatment. However, the lack of dopaminergic degeneration in this study, despite the chronic neuroinflammation, suggests that multiple inflammatory triggers may be required for LRRK2 mutation carriers to develop PD (Schildt et al., 2019). This is in contrast to acute responses to LPS treatment as no differences in cytokine levels and microglial changes were observed in G2019S mice compared to control mice, 90 min after LPS treatment (Schildt et al., 2019).

Taken together, pathogenic LRRK2 mutations appear to enhance the immune response during inflammatory conditions, such as chronic inflammatory diseases or acute infections, through immune cells from the periphery, which might in turn increase the susceptibility to develop PD (Figure 1). More evidence that LRRK2 is involved in such inflammatory conditions is discussed below.

## The Link Between LRRK2 and Inflammatory Diseases

LRRK2 appears to be closely linked to inflammatory bowel disease (IBD), which is a chronic inflammatory condition of the digestive tube that includes Crohn’s disease (CD) and ulcerative colitis (UC). As mentioned previously, IBD is an important risk factor to develop PD (Wan et al., 2018; Park et al., 2019; Weimers et al., 2019; Zhu et al., 2019) and evidence points to LRRK2 as a potential link between these apparently unrelated disorders. An association between the LRRK2 locus and IBD has been identified by GWAS (Liu et al., 2015; De Lange et al., 2017) and exome sequencing revealed that functional LRRK2 variants confer shared effects on the risk to develop CD and PD (Hui et al., 2018). Dendritic cells from CD patients were also shown to exhibit increased LRRK2 levels *in vitro*. However, the mechanisms whereby LRRK2 can increase the risk to develop IBD remain elusive as we only begin to understand its function in the gut (Takagawa et al., 2018). In a mouse model for UC-like pathology based on dextran sulfate sodium (DSS), transgenic mice overexpressing LRRK2 WT exhibited more severe colitis and increased proinflammatory cytokine production compared to littermate controls. LRRK2 kinase inhibitor treatment ameliorated the phenotype in transgenic and control mice (Takagawa et al., 2018), pointing to a role for the LRRK2 kinase activity in IBD pathogenesis. This is in line with the increased kinase activity of the LRRK2 variant N2081D, which is a risk variant for CD (Hui et al., 2018). In contrast, an independent study reported exacerbated colitis in LRRK2 deficient mice (Liu et al., 2011), indicating that the exact relation between LRRK2 and IBD requires further investigation.

Besides IBD, LRRK2 has also been studied in the context of peripheral infections, especially infections affecting the gastrointestinal tract. LRRK2 was reported to be protective against intestinal *Listeria monocytogenes* infection (Zhang et al., 2015). Similarly, LRRK2 appeared crucial for the antibacterial activity of macrophages during infection with *Salmonella typhimurium in vitro* (Gardet et al., 2010), which was confirmed *in vivo* using mice lacking LRRK2 (Gardet et al., 2010; Liu et al., 2017; Shutinoski et al., 2019). The protective effects of LRRK2 during bacterial infections seem to be mediated by its kinase activity since knockin mice expressing the G2019S variant were able to better control the infection, in contrast to mice expressing the kinase dead variant D1994S (Shutinoski et al., 2019).

The idea that LRRK2 may play a crucial role in the gut immune cells fits perfectly in the concept of the gut-brain-axis in PD. This connection between both organs could explain the intestinal symptoms in PD patients, the pattern of αSYN spreading described by the Braak stages and the link between systemic inflammation and neuroinflammation (Mulak and Bonaz, 2015; Santos et al., 2019). The involvement of LRRK2 in this gut-brain axis remains unclear but might be related to its function in immune cells given that LRRK2 is upregulated in intestinal immune cells of CD patients, where it might act as an IFN-γ target gene (Gardet et al., 2010). In addition, increased LRRK2 activity has been shown to alter bone marrow myelopoiesis and to have an impact on the intestinal immune system by suppressing Th17 cell differentiation (Park et al., 2017).

The role of LRRK2 has also been studied in inflammatory conditions affecting other organs. In line with the reported protective effects against intestinal infections, mouse pups carrying the LRRK2 G2019S mutation displayed reduced viral titers during reovirus (serotype 3TD)-induced encephalitis. Curiously, mutant LRRK2 induced an enhanced proinflammatory state that was protective during sepsis, but proved to be detrimental during encephalitis as it was linked to a higher mortality rate (Shutinoski et al., 2019). Intriguingly, opposite findings were described for *Mycobacterium tuberculosis*, with an enhanced bacterial control at early stages of infection in LRRK2 KO animals (Härtlova et al., 2018). LRRK2 was also found not protective in the autoimmune disease systemic lupus erythematosus, since LRRK2 levels in B cells positively correlated with disease severity (Zhang et al., 2019).

## Concluding Remarks

A better understanding of the etiology of PD will be key to find a disease-modifying therapy. However, it has become more and more clear that PD is a complex disease with different factors and pathogenic mechanisms. The multiple-hit hypothesis for PD suggests that an interaction between genetic and/or environmental risk factors is needed to trigger the disease and LRRK2 fits perfectly within this model. The G2019S LRRK2 mutation is highly prevalent and the most common cause of familial PD, but it has a surprisingly low penetrance of ∼25–40% (Goldwurm et al., 2007; Marder et al., 2015; Lee et al., 2017). Environmental triggers such as inflammation could synergize with the mutated protein to induce a detrimental effect. This idea is supported by the finding that inflammation is required to induce a PD phenotype in mice carrying mutant LRRK2 (Kozina et al., 2018). Additional multiple-hit studies to model LRRK2-PD might be instrumental to further unravel the pathogenic role of LRRK2.

The present knowledge of LRRK2 biology strongly points toward the immune system. Future studies focusing on peripheral immune cells are required, given the low LRRK2 expression in microglia and dopaminergic neurons (Gaiter et al., 2006; Melrose et al., 2006). It is intriguing to see that current studies point to opposite inflammatory effects of LRRK2 in the CNS vs. the periphery. While LRRK2 activity might be indirectly detrimental for the brain, it seems protective against some inflammatory insults in the periphery. LRRK2 kinase activity is positively linked to a pro-inflammatory response and might thus be beneficial to control peripheral pathogen infections. This might help explain the high prevalence of the LRRK2 G2019S mutation as an evolutionary advantage. Still, the protective effect of LRRK2 activity appears to depend on the specific pathogen. This apparent incongruency might be explained by differences in microorganisms or insults studied, and/or cell type-specific functions of LRRK2. One could argue that LRRK2 mediates different functions in different immune cells. This could clarify why LRRK2 protects against *S. typhimurium* and *L. monocytogenes* infections, which rely on the gut immune cells, but at the same time aggravates *M. tuberculosis* infections, which affects the respiratory system. This cell type/organ specificity is in line with the observation that LRRK2 KO mice are more susceptible to intestinal, but not systemic *L. monocytogenes* infections (Zhang et al., 2015).

The prominent role of LRRK2 in peripheral immune reactions that might lead to dysregulated microglial activity and thus contribute to neuroinflammation and neurodegeneration in PD can provide new therapeutic approaches. However, it also potentially complicates current therapeutic strategies relying on highly brain permeable LRRK2 kinase inhibitors. Although no side effects have been reported upon inhibition of LRRK2 in the brain, decreased systemic LRRK2 activity may induce a more permissive immune system, resulting in an inadequately controlled infection, dependent on the pathogen.

It will be interesting for future studies to identify in more detail the role of (mutant) LRRK2 during peripheral infections in terms of pathogen-specific mechanisms and the involvement of specific immune cells. These kind of studies will not only provide insight in the biology of inflammatory processes and thus support the development of specific therapies but might also help to understand how infections and environmental factors increase PD susceptibility.

## Author Contributions

DC and EL conceptualized, designed and drafted the manuscript. VB provided critical revisions of the manuscript.

## Conflict of Interest

The authors declare that the research was conducted in the absence of any commercial or financial relationships that could be construed as a potential conflict of interest.
